# Cooperation of neurotrophin receptor TrkB and Her2 in breast cancer cells facilitates brain metastases

**DOI:** 10.1186/s13058-017-0844-3

**Published:** 2017-04-26

**Authors:** Cecilia Choy, Khairul I. Ansari, Josh Neman, Sarah Hsu, Matthew J. Duenas, Hubert Li, Nagarajan Vaidehi, Rahul Jandial

**Affiliations:** 1 Division of Neurosurgery, Beckman Research Institute, City of Hope, 1500 E. Duarte Rd, Duarte, CA 91010 USA; 2 Irell & Manella Graduate School of Biological Sciences, City of Hope, Duarte, CA 91010 USA; 3 Department of Neurosurgery, Keck School of Medicine at University of Southern California, Los Angeles, CA 90089 USA; 4 Department of Preventive Medicine, University of Southern California, Los Angeles, CA 90089 USA; 5 Department of Immunology, Beckman Research Institute, City of Hope, Duarte, CA 91010 USA

**Keywords:** Breast cancer brain metastasis, Epidermal growth factor receptor 2 (Her2+), Tropomyosin-related kinase B (TrkB), Brain-derived neurotrophic factor (BDNF), Neurotrophins, Neural microenvironment, Astrocyte, Lapatinib, Cyclotraxin B, Glial cells, Neurotrophic factor

## Abstract

**Background:**

Patients with primary breast cancer that is positive for human epidermal growth factor receptor 2 (Her2+) have a high risk of developing metastases in the brain. Despite gains with systemic control of Her2+ disease using molecular therapies, brain metastases remain recalcitrant to therapeutic discovery. The clinical predilection of Her2+ breast cancer cells to colonize the brain likely relies on paracrine mechanisms. The neural niche poses unique selection pressures, and neoplastic cells that utilize the brain microenvironment may have a survival advantage.

**Methods:**

Tropomyosin-related kinase B (TrkB), Her2, and downstream targets were analyzed in primary breast cancer, breast-to-brain metastasis (BBM) tissues, and tumor-derived cell lines using quantitative real-time PCR, western blot, and immunohistochemical assessment. TrkB function on BBM was confirmed with intracranial, intracardiac, or mammary fat pad xenografts in non-obese diabetic/severe combined immunodeficiency mice. The function of brain-derived neurotrophic factor (BDNF) on cell proliferation and TrkB/Her2 signaling and interactions were confirmed using selective shRNA knockdown and selective inhibitors. The physical interaction of Her2-TrkB was analyzed using electron microscopy, co-immunoprecipitation, and in silico analysis. Dual targeting of Her2 and TrkB was analyzed using clinically utilized treatments.

**Results:**

We observed that patient tissues and cell lines derived from Her2+ human BBM displayed increased activation of TrkB, a neurotrophin receptor. BDNF, an extracellular neurotrophin, with roles in neuronal maturation and homeostasis, specifically binds to TrkB. TrkB knockdown in breast cancer cells led to decreased frequency and growth of brain metastasis in animal models, suggesting that circulating breast cancer cells entering the brain may take advantage of paracrine BDNF-TrkB signaling for colonization. In addition, we investigated a possible interaction between TrkB and Her2 receptors on brain metastatic breast cancer cells, and found that BDNF phosphorylated both its cognate TrkB receptor and the Her2 receptor in brain metastatic breast cancer cells.

**Conclusion:**

Collectively, our findings suggest that heterodimerization of Her2 and TrkB receptors gives breast cancer cells a survival advantage in the brain and that dual inhibition of these receptors may hold therapeutic potential.

**Electronic supplementary material:**

The online version of this article (doi:10.1186/s13058-017-0844-3) contains supplementary material, which is available to authorized users.

## Background

Metastasis, the spread of tumors from one organ to another, is responsible for the majority of cancer-related deaths [1]. The brain is unlike any other metastatic site due to its unique microenvironment [2, 3]. Within the brain, astrocytic glial cells normally function to support and maintain a balanced microenvironment for neuronal signaling and respond with reactive gliosis upon brain injury [4]. But when a neoplastic cell penetrates the brain’s barriers, astrocytes provide early and robust cellular responses by producing and releasing neurotrophins, such as brain-derived neurotrophic factor (BDNF) [5].

BDNF is a specific ligand for tropomyosin-related kinase B (TrkB), a tyrosine kinase receptor, which upon binding BDNF activates several pathways, including the phosphoinositide-3 kinase (PI3K) pathway [6, 7]. BDNF-TrkB signaling has been suggested to mediate cancer cell resistance to chemotherapy and promote growth of neoplastic cells. One way this has been suggested to occur is through autocrine signaling in primary tumor cells [8, 9]. Consistent with this, autocrine BDNF-TrkB signal transduction is associated with cell proliferation, differentiation, survival, and invasion [9–11]. However, the possible paracrine role of TrkB activation by BDNF in the brain microenvironment in metastatic Her2+ breast cancer needs to be further elucidated.

Although all breast cancer subtypes can metastasize to the brain, patients with Her2+ and TN primary breast tumors have a higher risk of developing brain metastasis [12–14]. Her2 is a transmembrane tyrosine kinase receptor that is enriched in more than 20% of breast cancers, and about 35% of Her2+ patients will develop brain metastases [15]. Furthermore, unlike patients with TN breast cancer, Her2+ patients develop brain metastases even while their systemic disease is well-controlled [16–18]. However, the molecular characteristics of Her2+ breast cancer cells that allow them to exploit the brain microenvironment for successful colonization remain largely unknown.

We hypothesized that neoplastic cells, including metastatic Her2+ breast cancer cells, in the brain microenvironment use BDNF produced and released by surrounding astrocytes. We found that astrocyte-derived BDNF in the neural niche is important for initial tumor cell colonization and proliferation of breast-to-brain metastasis (BBM) cells. In addition, microenvironmental BDNF-mediated activation of TrkB resulted in increased metastatic potential of Her2+ cells and formation of Her2-TrkB heterodimers on breast cancer cells. These results further support the idea that metastatic cells with adaptations that support growth in a foreign microenvironment have an advantage for colonizing the brain.

## Methods

### Breast cancer brain metastasis cell cultures and treatments

Fresh Her2+ and TN human tumor samples were acquired from patients undergoing resection of breast to brain metastases, in accordance with a City of Hope Institutional Review Board (IRB)-approved protocol (IRB #05091). A portion of each specimen was cultured in DMEM-F12 (Life Technologies) supplemented with 10% fetal bovine serum (FBS), 1% glutamax, and 1% Anti-Anti (Life Technologies) in collagen-coated (Life Technologies) T75 flasks to derive low-passage primary cell lines (COH-BBM1 (BBM1), and COH-BBM2 (BBM2) and COH-BBM3 (BBM3)) [19]. Established breast cancer cell lines MDA-MB-361 (361), BT474 and SkBr3 cells were also cultured in the aforementioned DMEM-F12 in T75 flasks. All cells were maintained at 37 °C and 5% CO_2_.

To obtain conditioned medium from astrocytes and fibroblasts, cells were grown in serum-free DMEM for 24 h before collecting and purifying the medium by centrifugation (4000 × g, 15 minutes). For growth in conditioned medium, BBM cells were first grown for 24 h in serum-free DMEM. The growth medium was removed and the cells were washed once with fresh DMEM before adding the conditioned medium. Cells were grown for differing amounts of time in the conditioned medium, as indicated for specific experiments. For treatment with BDNF, lapatinib, cyclotraxin B, XL 147 or GDC0941, cells were grown overnight in serum-free mediuma before treatment with BDNF, lapatinib and cyclotraxin B for various time periods. For details on materials and methods please see Additional file 1.

### Real-time PCR and western blot analysis

Total RNA was extracted using Trizol (Invitrogen) and treated with RNase-free DNase (Qiagen) according to manufacturer instructions. To analyze expression of TrkB and Her2, cDNA was synthesized using the iScript reverse transcription kit (Bio-Rad). Real-time quantification was done using specific primers and SYBR select master mix for CFX (Applied Biosystems). Control PCR reactions were done using glyceraldehyde-3-phosphate dehydrogenase (GAPDH) and/or actin-specific TaqMan probe.

For western blot analysis, total cell lysates were prepared in protein lysis buffer (50 mM TrisHCl, pH 7.5; 100 mM NaCl; 1% Triton X-100; 1 mM EDTA; 1 mM EGTA, 50 mM β- glycerophosphoran, 1 mM dithiothreitol, 1 mM phenylmethanesulfonyl fluoride; 2 mM sodium orthovanadate, 10 μg/mL aprotinin; 10 μg/mL leupeptin and 10 μg/mL pepstatin A) by incubating cells (20 minutes, 4 °C) followed by centrifugation (15000 × g, 15 minutes, 4 °C). The protein extracts were analyzed by western blot using antibodies specific to TrkB and p-TrkB (Abcam); Her2 and phosphorylated (p)-Her2 (Millipore); phosphorylated protein kinase B (p-AKT), phosphorylated mitogen-activated protein kinase (p-MAPK), and phosphorylated phosphoinositide-specific phospholipase y (p-PLCy; Cell Signaling). Actin and α-tubulin were used as loading controls.

### Electron microscopy

Control BBM1 cells or BBM1 cells treated with 25 ng/mL BDNF were collected and cryo-fixed in a high-pressure freezer. Sections (~70 nm thick) were cut using a Leica Ultra cut UCT ultramicrotome with a diamond knife and placed on mesh nickel electron microscope grids. The grids were stained with 2% uranyl acetate in 70% ethanol for 1 minute followed by Reynold’s lead citrate staining for 1 minute. For post-embedding immuno-labeling, antigens were detected with 10 nm (for TrkB) or 20 nm (for Her2) colloidal gold conjugated secondary antibody. For pre-embedding immuno-labeling, cells were incubated with TrkB antibody overnight and then incubated with secondary antibody conjugated with 1.4 nm nanogold at room temperature. Cells were fixed (0.2% glutaraldehyde in PBS, room temperature, 10 minutes) and developed for 5 minutes with an HG silver enhancement kit (Nanoprobes). After the cell pellet was sectioned, post-embedding labeling was performed with the Her2 antibody overnight. Labeled sections were incubated with colloidal gold conjugated secondary antibody (15 nm) for 1 h. Images were acquired at × 11000 magnification. Cells were imaged and receptors were quantified by analyzing 200 images per receptor (100 images from the pre-embedding method and 100 images analyzed from the post-embedding method).

### Co-immunoprecipitation

Cells were homogenized with lysis buffer in the presence of protease and phosphatase inhibitors for 15 minutes on ice as described [20]. Protein was harvested by centrifugation (13000 × g, 10 minutes, 4 °C). The protein extract (750 μg) was incubated overnight at 4 °C with Protein G agarose beads (Invitrogen) pre-conjugated with primary antibody (4 μg). Agarose beads conjugated with IgG were used as a control. Beads were washed five times with high salt, low salt, LiCl, and TE buffers containing 0.04% NP4, by incubating for 5 minutes at 4 °C followed by centrifugation (750 × g, 3 minutes). Beads were then resuspended in loading buffer (95% Laemmli Buffer, 5% β-mercaptoethanol).

The protein extracts (input) and the immunoprecipitates were analyzed by western blot using p-Her2 (Life Technologies) and p-TrkB (Abcam) primary and horseradish peroxidase (HRP)-conjugated secondary antibodies. Actin antibody was used as a quantitative control for the inputs. Membranes were developed using Supersignal West Pico Chemiluminescent Substrate and analyzed under Li-Cor scanning (Li-Cor).

### In vivo xenografts

All mouse studies were conducted in accordance with a City of Hope Institutional Animal Care And Use Committee-approved protocol (COH IACUC #10044). For evaluating tumor growth and survival, BBM1 or BBM1-KD and BBM2 and BBM2-KD cells were transduced with a firefly luciferase lentivirus (firefly luciferase-Zs-Green; Addgene) and selected based on green-fluorescent protein (GFP) expression for injection into non-obese diabetic (NOD)-severe combined immunodeficiency (SCID) mice. Firefly luciferase-labeled BBM1 or BBM1-KD cells and BBM2-BBM2-KD (200,000 cells) in 2 μL medium for cranial or 20 μL medium for mammary fat pad (MFP) were injected into the respective sites. Tumor growth was monitored weekly by BLI on a Xenogen Imaging System with Living Image Software for data acquisition (Xenogen Corp). For treatment with PI3k inhibitor, the MFP-implanted animals were observed by BLI for tumor appearance. At 10 days post implantation of BBM1 cells, the tumor-bearing animals were orally dosed with GDC-0941(150 mg/kg), formulated in 0.5% methylcellulose and 0.2% Tween-80 (MCT) at a 3-day interval. At the conclusion of the experiments, mice were euthanized and the injection and metastasis sites were dissected and placed in formalin (Fisher Scientific).

For in vivo studies for competitive xenografts, BBM1 cells were transduced with firefly luciferase-tagged with red-fluorescent protein (RFP) (BBM1-FF-RFP) and BBM1-KD cells were transduced with Renilla tagged with GFP (BBM1-KD-Ren-GFP). GFP+ or RFP+ populations were collected by fluorescence-activated cell sorting (FACS). Cells were injected intracardially (50,000 cells) or into the MFP (100,000 cells) of NOD-SCID mice in a BBM1: BBM1-KD ratio of 1:1 or 1:4. Tumor growth was monitored weekly by BLI. At the conclusion of experiments, mice were euthanized and the primary and metastasis sites were dissected and placed in formalin (Fisher Scientific).

### Statistical analyses

Data represented in the figures are the mean values ± standard error. Statistical significance was assessed with one-way or two-way analysis of variance (ANOVA) and the Bonferroni multiple comparison test (GraphPad Prism). Results were considered statistically significant for *p* values <0.05, denoted as *p < 0.05, **p < 0.01, ***p < 0.001 and ****p < 0.0001.

## Results

### Her2+ BBMs express high levels of phosphorylated TrkB

**Fig. 1:**
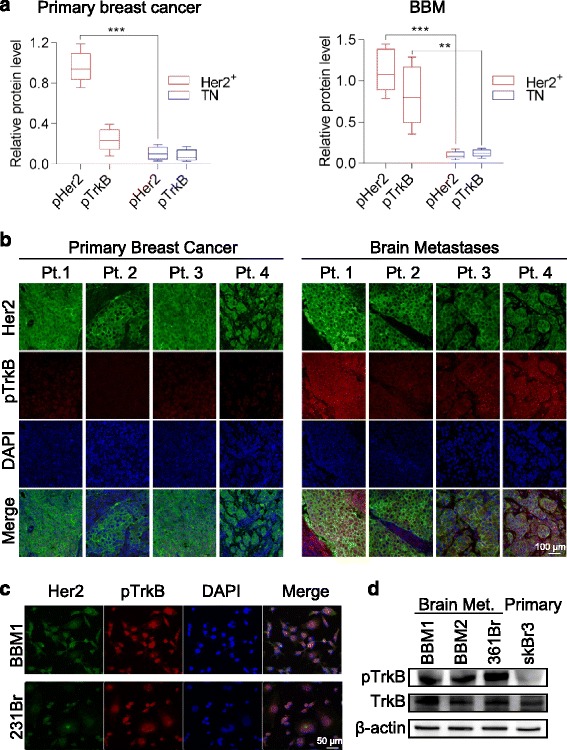
Human epidermal growth factor receptor 2 (*Her2*)-positive (*Her2+*) breast cancer brain metastasis (*BBM*) cells express high levels of phosphorylated neurotrophin receptor tropomyosin-related kinase B (*p-TrkB*). **a** Quantification of Her2 and TrkB in resected Her2+ (*n* = 8) and triple negative (*TN*) (*n* = 4) primary breast cancer tissue (*left*) and Her2+ (*n* = 3) and TN (*n* = 3) breast-to-brain metastases (*p < 0.05, **p < 0.01, ***p < 0.001; *bars* indicate SEM). **b** Representative immunofluorescence staining of primary breast cancer (*left*, *n* = 4) and breast-to-brain metastasis tissue (*right*, *n* = 4) from four patients (*Pt*). Tissue was stained for Her2 (*green*) and p-TrkB (*red*). Nuclear counter staining was done with 4',6-diamidino-2-phenylindole (*DAPI*, *blue*). **c** Immunofluorescence staining of p-TrkB in BBM1 and 231Br brain metastasis breast cancer cell lines. **d** Western blot analysis of p-TrkB and total TrkB in BBM1, BBM2, 361, and SkBr3 cells

Reactive astrocytes in central nervous system (CNS) malignancies release neurotrophins and bone morphogenic proteins. We recently found that bone morphogenic protein-2 (BMP-2), a ligand that promotes astrocytic differentiation, enhances the ability of BBM tumors to engraft and colonize the brain [21]. Neurotrophins are growth factors that promote neuronal differentiation and survival [22, 23]. To investigate the importance of neurotrophin receptors in BBM, we first compared p-TrkB in tumor tissues derived from Her2+ versus TN patient primary tumors (12 Her2+ and 12 TN) and from BBM (6 Her2+ and 3 TN). Although primary Her2+ breast tumors expressed significantly higher levels of Her2 as compared to TN tumors (*p* < 0.05), levels of p-TrkB were low, regardless of tumor subtype. In BBM tissue, however, levels of both p-Her2 and p-TrkB were significantly higher (*p* < 0.05) in Her2+ as compared to TN tumors (Fig. 1a). Quantitative real-time (qRT)-PCR analysis showed subtle differences in TrkB transcript levels between Her2+ and TN primary breast cancer and BBM cells (Additional file 2: Figure S1). Immunofluorescence staining confirmed greater p-TrkB expression in Her2+ BBM tumor tissue samples relative to Her2+ primary breast tumor samples (Fig. 1b). In addition, p-TrkB co-localized with Her2 in patient-derived BBM cells (Fig. 1c and Additional file 2: Figure S1b). More p-TrkB was also observed in cultured brain metastasis cells (BBM1 and BBM2) as compared to TN brain metastasis (MDA-MB-231Br ((231 Br)) and Her2+ primary breast cancer cell lines (SkBr3) (Fig. 1c and Additional file 2: Figure S1b). Immunoblotting experiments confirmed the higher levels of p-TrkB in BBM cells versus primary Her2+ breast cancer cells (Fig. 1d). To clarify whether the high TrkB expression in BBM cells is influenced by the brain microenvironment, BBM1 cells transduced with firefly luciferase-Zs-Green were xenografted into the MFP of NOD-SCID mice, and tumor growth was monitored by bioluminescence imaging (BLI). These tumors subsequently metastasized to the brain, bone, liver, and lung, but the brain metastases had significantly (*p* < 0.05) greater TrkB mRNA levels relative to metastases in other regions (Additional file 2: Figure S1c). This suggests the brain microenvironment uniquely promotes TrkB expression in colonizing Her2+ breast cancer cells.

### BDNF increases proliferation of Her2+ BBM cells

**Fig. 2:**
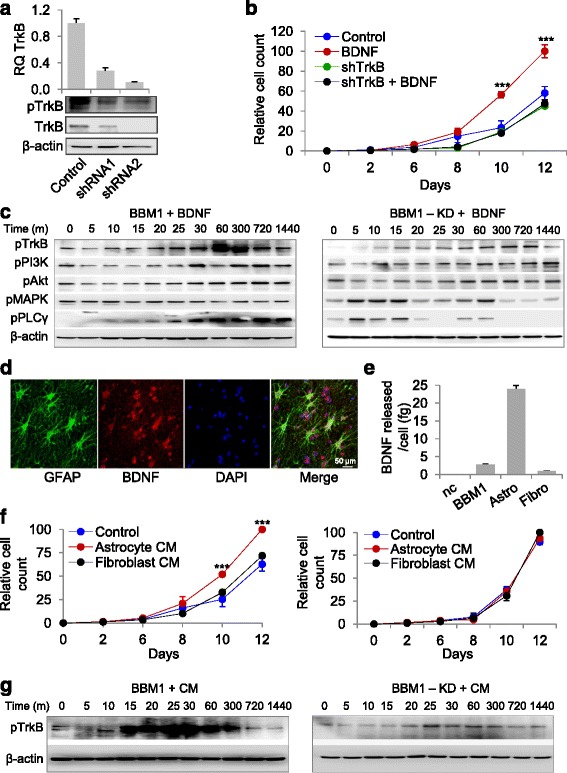
Exogenous and astrocyte-released brain-derived neurotrophic factor (*BDNF*) binds Tropomyosin-related kinase B (*TrkB*) leading to increased tumor cell proliferation. **a** Real-time PCR and western blot analysis of TrkB knockdown with two different short hairpin (*sh*)RNAs in BBM1 cells. Control was non-transduced BBM1 cells. **b** Effect of exogenous BDNF (25 ng/mL) on BBM1 (BDNF) and TrkB knockdown BBM1 (*shTrkB*) cell proliferation over 12 days in vitro*.* Control was non-treated cells (*n* = 3; ****p < 0.0001; *bars* indicate SEM). **c** Western blot analysis of PI3K pathway activation by exogenous BDNF (25 ng/mL) over 24 h in BBM1 and TrkB knockdown BBM1 (BBM1-KD) cells. **d** Immunohistochemical staining of glial fibrillary acidic proteins (*GFAP*) and BDNF in the peritumoral region in a human breast cancer brain metastasis (*BBM*) specimen. GFAP (*green*), BDNF (*red*), DAPI (*blue*). **e** ELISA quantification of BDNF released from BBM1 cells, astrocytes, and fibroblasts (*nc* medium only; ****p < 0.0001)*.* **f** Proliferation of BBM1 (*left*) and TrkB knockdown BBM1 (BBM1-KD) (*right*) cultured with control DMEM (*Control*), astrocyte-conditioned medium (*Astrocyte CM*), or fibroblast-conditioned medium (*Fibroblast CM*) for 12 days in vitro (*n* = 3; **p < 0.01, ****p < 0.0001; *bars* indicate SEM). **g** Phosphorylation of TrkB in BBM1 and TrkB knockdown BBM1 (*BBM1-KD*) cells grown in astrocyte-conditioned medium (*CM*)

To test whether BDNF-TrkB signaling promotes brain metastasis, we established lentiviral short hairpin (sh)RNA-mediated knockdown of TrkB in BBM1 (BBM1-KD and BBM1-KD2) and MDA-MB-361(361-KD and 361-KD2) cells. Knockdown was confirmed by qRT-PCR and western blot analysis (Fig. 2a, Additional file 2: Figure S2a). Culturing these cells in medium containing BDNF (25 ηM) for 12 days led to increased cell proliferation in BBM1 and 361 TrkB-expressing cells (*p* < 0.05), but not in TrkB-knockdown cells (Fig. 2b, Additional file 2: Figure S2b). Culturing BBM1 and 361 cell lines (regardless of TrkB expression status), in the BDNF-containing medium for 24 h increased expression of p-TrkB, p-PI3K, and p-AKT (Fig. 2c, Additional file 2: Figures S2c, S3) as expected [6]. In contrast, BDNF stimulation failed to significantly activate PI3K/AKT signaling in TrkB-knockdown cells (Fig. 2c, Additional file 2: Figure S2c, Figure S3). This led us to conclude that activation of the PI3K/AKT signaling pathway after stimulation with BDNF increased proliferation of BBMs.

### Astrocyte-derived BDNF increases proliferation of Her2+ BBM cells

Previous research suggests that autocrine signaling in breast cancer cells is responsible for BDNF-TrkB-induced metastasis [9]. Interestingly, our previous studies using in vivo MFP xenograft models showed low BDNF gene expression in brain metastases (Additional file 2: Figure S4), despite high TrkB expression (Additional file 2: Figure S1). This suggested BBM cells can also use the brain’s endogenous microenvironmental BDNF instead of generating their own. Because the brain microenvironment contains high concentrations of BDNF, we proposed that paracrine signaling between astrocyte-derived BDNF and TrkB on breast cancer cells supports successful initial brain colonization.

Previously, we reported the importance of cross-talk between breast cancer cells and adjacent astrocytes in the brain microenvironment [24]. Consistent with this, our patient-derived tumor specimens showed BDNF expression in astrocytes in the peritumoral region (Fig. 2d), whereas we saw similar levels of intracellular BDNF in all examined tumor-derived and non-tumor cell lines (BBM1, 361, astrocytes, and fibroblasts) (Additional file 2: Figure S5a). However, cultured astrocytes released approximately 20-fold more (*p* < 0.05) BDNF into the conditioned media as compared to the other cell types (Fig. 2e, Additional file 2: Figure S5b). To clarify the proliferative role of paracrine BDNF-TrkB interactions, we cultured BBM1 or 361 cells in astrocyte or fibroblast-conditioned medium (CM) and counted cells every two days. BBM1 and 361 cells showed increased proliferation when cultured in astrocyte CM relative to control media or fibroblast CM (Fig. 2f, Additional file 2: Figure S5c). The modest increase in proliferation for cells cultured with fibroblast CM may be due to growth factors in the fibroblast CM. Moreover, TrkB-knockdown cells cultured in astrocyte CM did not show increased proliferation, suggesting that paracrine TrkB-BDNF signaling in the brain microenvironment is advantageous for BBM proliferation. TrkB phosphorylation increased when BBM1 cells were cultured in astrocyte CM; this is an effect that was abrogated in the TrkB-knockdown cell lines (Fig. 2g, Additional file 2: Figure S5d). Indeed, treatment of the cells with XL147 and GDC0941 inhibitors of PIK3 a TrkB downstream kinase also suppressed astrocyte-CM-induced cell proliferation (Additional file 2: Figure S5e-f). These results indicate that release of astrocyte-derived BDNF facilitates effective brain colonization by breast cancer cells.

### TrkB-knockdown in Her2+ BBMs disrupts colonization and metastatic efficiency in the brain

**Fig. 3:**
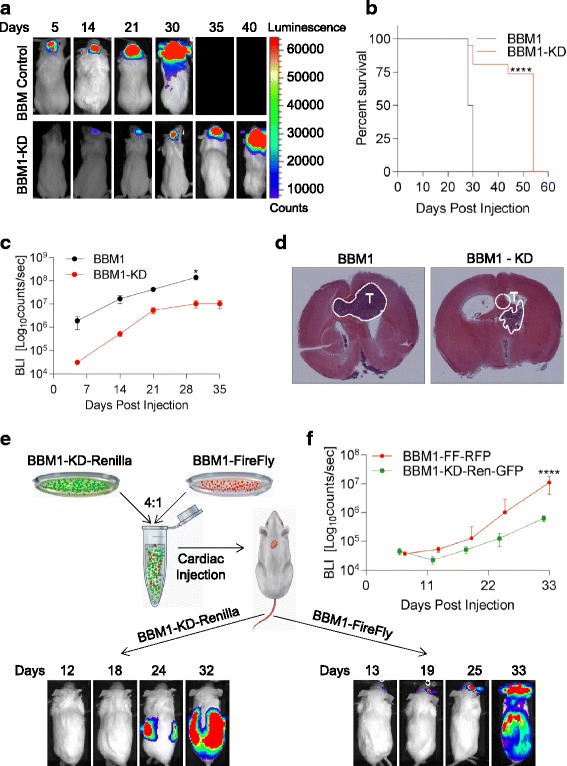
Tropomyosin-related kinase B (TrkB) knockdown disrupts colonization and metastatic efficiency of breast cancer brain metastasis (*BBM*) xenografts. **a** Representative bioluminescence imaging (*BLI*) of intracranially injected BBM1 or BBM1-knockdown (*BBM1-KD*) cells in NOD-SCID mice. **b** Survival curve of mice injected intracranially with BBM1 or BBM1-KD cells (****p < 0.0001). **c** Tumor growth over 35 days (*n* = 6; *p < 0.05; *bars* indicate SEM). **d** Hematoxylin and eosin stained brain sections from mice euthanized 28 days after intracranial injection of BBM1 or BBM1-KD cells: tiled images (×5 magnification). *White perforated lines* indicate locations of the tumor. **e** The schema for BBM1 and BBM1-KD co-injection study (*top*). Representative BLI images of mice that received cardiac injections (*bottom*). **f** BLI of brain metastases arising from BBM1 cells in mice that received cardiac injections shown in **e** (*n* = 6; ****p < 0.0001; *bars* indicate SEM). *Ren* renilla, *RFP* red fluorescent protein, *GFP* green fluorescent protein

To examine the importance of TrkB on tumor cell colonization in vivo, firefly luciferase-labeled BBM1 or BBM1-knockdown (BBMI-KD) and BBM2 or BBM2-KD cells were injected intracranially into NOD-SCID mice. BLI analysis of the brain indicated that tumors arising from both BBM1-KD and BBM2-KD cells had slower growth kinetics compared to control cells (Fig. 3a, c, d and Additional file 2: Figure S6a) and their growth reached a plateau around days 28 (BBM1) and 33 (BBM2) after injection. In addition, mice bearing BBM1-KD or BBM2-KD tumors had approximately twofold increase in overall survival as compared to mice injected with TrkB-expressing BBM1 or BBM2 cells (Fig. 3b and Additional file 2: Figure S6b).

We then explored in Her2+ BBM cells the potential advantage TrkB expression may give for metastatic efficiency and brain colonization by co-injecting BBM1 cells (BBM1-FF-RFP) with BBM1-KD cells (BBM1-KD-Ren-GFP) via intracardiac or MFP delivery. Following intracardiac co-injection of BBM1 and BBM1-KD cells, the TrkB+ tumor cells established systemic metastasis, including within the brain (Fig. 3e, Additional file 2: Figure S7a-d). The TrkB-negative (TrkB-) cells also formed metastases in various organs, but they did not produce significant brain metastases in NOD-SCID mice. Further analysis showed that inhibition of PI3K a downstream kinase of TrkB signaling, with GDC0941, also suppressed overall metastasis of intracardially injected BBM1 cells. However, GDC0941 does not affect brain tropic metastasis efficiency (Additional file 2: Figure S7e-f). These results suggest that expression of TrkB is necessary for breast cancer cells to successfully form metastatic colonies in a BDNF-enriched brain microenvironment.

### Her2 and TrkB co-localize upon BDNF administration in BBM cells

**Fig. 4:**
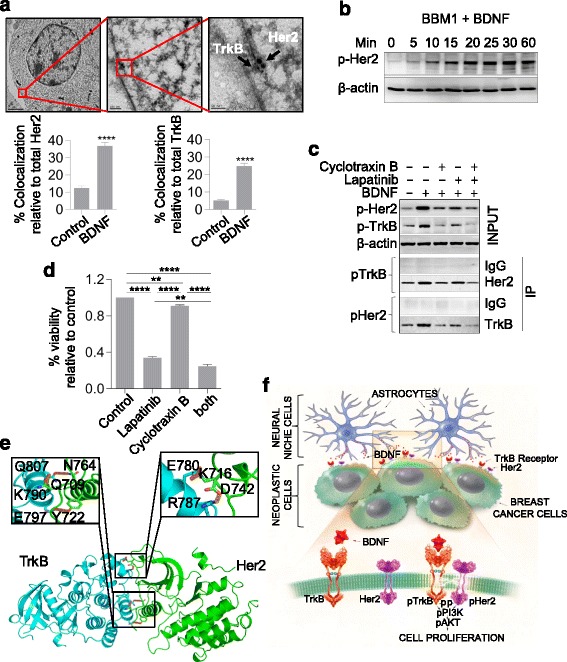
Tropomyosin-related kinase B (*TrkB*) and human epidermal growth factor receptor 2 (*Her2*) heterodimerize and activate upon brain-derived neurotrophic factor (*BDNF*) administration. **a** Representative post-embedding electron microscopy image (*top*) of TrkB and Her2 clustering at the cell membrane upon BDNF treatment; *t* = 30 minutes, TrkB = 10 nm, Her2 = 20 nm. Quantification (*bottom*) of co-localized Her2 or TrkB receptors relative to total Her2 or TrkB receptors, respectively (*bars* indicate SEM). **b** Western blot analysis of Her2 activated by exogenous BDNF over 1 h in BBM1 cells. **c** Co-immunoprecipitation of TrkB and Her2 from BBM1 cells grown in the presence of lapatinib (50 μM) and/or cyclotraxin B (20 μM). Her2 and TrkB immunoprecipitations were analyzed by western blotting with anti-TrkB and anti-Her2 antibodies, respectively. **d** Molecular model of TrkB (*left*) and Her2 (*right*). Insets indicate amino acids on TrkB and Her2 involved in possible hydrogen bonding. **e** Quantification of viable BBM1 cells grown in the presence of Her2 and TrkB inhibitors lapatinib (50 μM) and cyclotraxin B (20 μM), respectively. **f** Predicted model of paracrine signaling between breast cancer cells and the brain microenvironment. Her2 receptor (*orange*) and TrkB receptor (*purple*) on the cell surface membrane (*green*) are phosphorylated upon binding of BDNF (*red*) secreted by surrounding astrocytes (*blue*)

The Her2 receptors in breast cancer heterodimerize typically with epidermal growth factor receptor (EGFR) family members upon activation [24]. Therefore, we investigated the potential interactions between Her2 and other tyrosine kinase receptors in BBM cells. Flow cytometry experiments using antibodies that recognize the extracellular domains of the Her2 and TrkB receptors showed that approximately half of BBM1 and SkBr3 cells co-expressed both receptors (Additional file 2: Figure S8). Electron microscopy further revealed subcellular co-localization of Her2 and TrkB on BBM1 and SkBr3 cell membranes. We found that stimulating the cells with BDNF resulted in fourfold and sixfold increased co-localization of Her2 and TrkB, respectively, compared to cells cultured without BDNF (Fig. 4a, Additional file 2: Figure S9). We used immunofluorescence to confirm increased co-localization of TrkB and Her2 following BDNF stimulation (Additional file 2: Figure S9). Thus, BDNF stimulation promotes physical interaction between Her2 and TrkB receptors in BBMs.

### Simultaneous inhibition of Her2 and TrkB reduces survival of Her2+ BBM cells

To test whether co-localization of TrkB and Her2 regulates activation of downstream effector pathways, we incubated BBM1 cells with BDNF and quantified the temporal activation of Her2 (p-Her2). We found that BDNF stimulation resulted in acute activation of Her2 (Fig. 4b). Co-immunoprecipitation experiments showed that TrkB and Her2 receptors interact in BBM1 cells (Fig. 4c). To understand the functional relevance of TrkB and Her2 phosphorylation in establishing interactions between the two receptors, we used well-established inhibitors to inhibit both TrkB and Her2 signaling. Incubation of BBM1 cells with cyclotraxin B (TrkB-specific inhibitor) and lapatinib (Her2 inhibitor) alone, or in combination, attenuated BDNF-mediated activation of Her2 and TrkB and decreased the interaction between the two receptors (Fig. 4c).

To clarify the functional relevance of interaction between Her2 and TrkB, we measured the cytotoxicity of dual inhibition of the receptors. Co-incubation of BBM1 cells with cyclotraxin B and lapatinib for 48 h led to significantly fewer viable cells (*p* < 0.05) as compared to incubation with a single inhibitor or no inhibitor (Fig. 4d). Fewer viable cells were also observed upon treatment with lapatinib (*p* < 0.05), and cyclotraxin B (*p* < 0.05) compared to untreated control.

In silico 3D analysis of Her2 and TrkB crystal structures supported our finding that these receptors can physically interact (Fig. 4e). Protein molecular modeling predicted five amino acids in the kinase region of TrkB (Q807, K790, E797, E780, R787) with the potential to interact with Her2 (N746, Q709, Y722, K716, D742) through hydrogen bonding (Fig. 4e).

## Discussion

Breast cancer patients often develop brain metastases years, even decades, after their initial diagnosis [25, 26]. The presence of circulating tumor cells (CTCs) in patients during early stages of breast cancer and the ability of only a small population of CTCs to traverse the blood–brain barrier suggest that colonization within the brain is a rate-limiting step in the metastatic cascade [27]. We hypothesized that expression and activation of the neurotrophic receptor TrkB on metastatic breast cancer cells may provide a survival advantage to Her2+ breast cancer cells that are exposed to the neural niche. We found that Her2+ breast cancer cells have increased TrkB protein levels in response to astrocyte-derived BDNF stimulation, resulting in the formation of TrkB and Her2 heterodimers and brain metastases.

Increased levels of BDNF ligand have been reported in primary breast tumors as compared to normal tissue [28], and neurotrophin receptors are expressed in various extracranial malignancies [29]. Autocrine mechanisms involving neurotrophins have been suggested to aid various extracranial malignancies due to decreased expression of neurotrophins in the surrounding microenvironment [9, 29–31]. Our in vivo data demonstrated high BDNF mRNA expression in primary MFP tumors. Because breast tissue lacks microenvironmental BDNF, activation of neurotrophin receptors likely uses an autocrine mechanism. In addition, the fact that primary breast cancer cells express non-phosphorylated TrkB (Fig. 1), suggests some adaptations are inherent to primary tumor cells before metastasis. Whether these adaptations also steer metastatic organotropism is unclear, but genes that may predict a primary cell’s future metastatic site need to be explored further [32, 33].

Astrocytes uniquely release exosomes that make colonizing tumor cells lose the phosphatase and tensin homolog (PTEN) gene [34], a negative regulator of the PI3K/AKT signaling pathway. We found that release of astrocyte-derived BDNF triggered activation of the PI3K/AKT pathway in BBM cells, providing an explanation for the increased survival advantage. Levels of astrocyte-derived neurotrophins, including BDNF, are increased after brain injury and brain metastases, including breast cancer brain metastases [29, 30, 35–37]. Our work provides further evidence that astrocytes represent a key factor in brain colonization by metastasizing tumor cells.

The heterogeneity of splice variants is an important factor for TrkB receptor function. Multiple splice variants of TrkB are expressed in different cell types, and extracellular conditions may affect structural variations, which may be further explored by continued in vivo or 3D modeling studies. Although we focused on phosphorylated TrkB receptors, a previous study reported that TrkB.T1, an isoform that lacks the kinase domain, is the predominant TrkB receptor involved in breast cancer metastasis [9]. In addition, our data indicate that TrkB receptor phosphorylation is necessary for response to microenvironmental BDNF and for successful brain colonization.

Trk family members are also associated with members of the EGFR family, which includes the Her2 receptor [37]. Given the clinical predilection of Her2-amplified tumors to develop brain metastases, we explored interactions between Her2 and TrkB because TrkB heterodimerization with EGFR could increase proliferation and migration in ovarian cancer cells [38]. Co-immunoprecipitation and co-localization experiments showed associations between the TrkB and Her2 receptors (Fig. 4), and these associations increased after BDNF administration. The identification of tyrosine kinase receptors that heterodimerize with Her2 could provide new targets for therapy, particularly in the context of the right microenvironment. Our data show that dual inhibition of Her2 and TrkB receptors decreases BBM cell viability, possibly by inhibiting BDNF-induced activation of Her2 and TrkB (Fig. 4c, e).

## Conclusions

Collectively our results suggest that BDNF signaling to BBM cells supports colonization and tumor growth in the brain microenvironment (Fig. 4f). TrkB may represent a potential therapeutic target for treating or preventing brain metastases in patients with select subtypes of breast cancer, such as those with Her2+ disease. Further, the molecular cooperation between TrkB and Her2 receptors may partially explain the clinical predilection of Her2+ breast cancer patients to develop brain metastases and warrants further preclinical investigation. Effective clinical translation will most likely require targeting of the neoplastic “seed” and perturbation of the microenvironmental “soil.”

## Additional files

Additional file 1: Supplementary text - details on materials and methods. (PDF 111 kb)

Additional file 2: Figure S1. Expression of TrkB in various cells and tissue. **Figure S2.** Analysis of TrkB knockdown with two different TrkB shRNAs in patient-derived MDA-MB-361 cells (361-KD and 361-KD2). **Figure S3.** Western blot analysis of protein extracts from patient-derived 361 cells and 361 cells transduced with TrkB shRNA-1 (361-KD) or TrkB shRNA-2 (361-KD2) and cultured with BDNF. **Figure S4.** Real-time PCR quantification of BDNF expression in different metastasis sites. **Figure S5.** Western blot analysis of intracellular BDNF in BBM1 cells, 361 cells, astrocytes, and fibroblasts and proliferation of 361 cells and TrkB-specific shRNA1 (361-KD) and shRNA2 (361-KD2) transduced cells grown in astrocyte or fibroblast conditioned medium (CM). **Figure S6.** TrkB knockdown disrupts the metastatic efficiency of BBM2 xenografts. **Figure S7.** TrkB knockdown disrupts colonization and metastatic efficiency in BBM xenografts. **Figure S8.** TrkB and Her2 are co-expressed in BBM and breast cancer cells. **Figure S9.** Immunocytochemical staining of BBM1 and TrkB knockdown (BBM1-KD) cells treated with BDNF over different times. (PDF 1352 kb)

## Abbreviations

BBM: Breast cancer brain metastasis
BDNF: Brain-derived neurotrophic factor
BLI: Bioluminescent imaging
BMP-2: Bone morphogenic protein-2
CM: Conditioned medium
CNS: Central nervous system
CTCs: circulating tumor cells
DAPI: 4′,6-Diamidino-2-phenylindole
DMEM: Dulbecco’s modified Eagle’s medium
EGFR: epidermal growth factor receptor
ELISA: enzyme-linked immunosorbent assay
ERBB2: Epidermal growth factor receptor 2
FBS: Fetal bovine serum
GAPDH: Glyceraldehyde-3-phosphate dehydrogenase
GFP: Green-fluorescent protein Her2, Human epidermal growth factor receptor 2
MFP: Mammary fat pad
NOD: Non-obese diabetic
p-Atk: Phosphorylated protein kinase B
PBS: Phosphate-buffered saline
p-MAPK: Phosphorylated mitogen-activated protein kinase
p-PLCy: Phosphorylated phosphoinositide-specific phospholipase y
qRT-PCR: quantitative real-time polymerase chain reaction
RFP: Red-fluorescent protein
SCID: Severe combined immunodeficiency
shRNA: Short hairpin RNA
TN: Triple negative
TrkB: Tropomyosin-related kinase B
