# Supplementary material for: Cooperation of neurotrophin receptor TrkB and Her2 in breast cancer cells facilitates brain metastases
Source: Breast Cancer Res. 2017 Apr 26;19:51. doi: 10.1186/s13058-017-0844-3 (PMC5406906; doi:10.1186/s13058-017-0844-3)
Supplement: Supplementary file 1 — Supplementary text - details on materials and methods. (PDF 111 kb) [file 13058_2017_844_MOESM1_ESM.pdf]

Supplemental Information

Cell Culture

TrkB knockdown cell lines (i.e., BBM1-KD and 361-KD cells) were prepared by transducing BBM1 or 361 cells with a lentivirus encoding shRNA for TrkB. Transduction was done in the presence of 4 μg/mL Polybrene (Sigma). The culture media was replaced 24 h after transduction with fresh media, and 48 h after addition of fresh media, 1.5 μg/mL puromycin (Sigma) was added to select for puromycin-resistance. Knockdown of TrkB expression was confirmed by western blot analysis.

qRT-PCR

The most common sites for metastatic disease (brain, bone, liver, and lung) in breast cancer and the primary mammary fat pad (MFP) tumors were dissected from *in vivo* experiments (see below). RNA was isolated using a RNA extraction kit and following the organ lysis protocol (Qiagen). Each RNA sample (500 ng) was converted to cDNA using the RT^2 First Strand Kit (Qiagen) and combined with the RT^2 SYBR Green qPCR Mastermix (Qiagen). Equal volumes (25 μL) of this mixture were loaded into each well of neurotrophin and neurotrophin receptor RT^2 Profiler PCR array plates (Qiagen). qPCR was performed using an iQ5 Real-time PCR Detection System (Bio-Rad). Expression levels were normalized to GAPDH and actin.

Cell Proliferation

For cell proliferation assays, 120 cells were plated per well in a 12-well plate and incubated at 37ºC and 5% CO_2 (day 0). BDNF (25 ng/mL), astrocyte conditioned media

(CM), or fibroblast CM was applied to the wells. Cells were collected and counted every 2 days during 12-day proliferation assays. Each sample was run in triplicate.

Immunohistochemistry of Formalin-Fixed Tissue

Patient BBM tissue specimens obtained as described in the main text were formalin-fixed and embedded in paraffin. Sections (10 μm) were cut onto slides and baked for 3 h. Wax was removed with xylene and the tissue was hydrated with a gradient of ethanol followed by PBS washes. For antigen retrieval, slides were boiled in 10 mM sodium-citrate buffer (Tris Sodium Citrate (J.T.Baker), pH 6.0) for 1 h. Slides were permeabilized in 0.3% Tween-20 at 37°C for 45 min and blocked (1% bovine serum albumin (Sigma), 10% normal goat serum (, Invitrogen), 0.3 M glycine) at room temperature for 1 h. Primary antibodies in a carrier of 1.5% NGS and 1% BSA in PBS were applied overnight at 4ºC. Fluorescent secondary antibodies (Jackson ImmunoResearch Laboratories) were applied the following day for 1 h at room temperature in the dark. Coverslips (Warner Instruments) were placed on slides and mounted with Immunogold with DAPI (Life Technologies). Slides were analyzed on a Carl Zeiss LSM Confocal Microscope and Z-stack images were collected and collapsed. Antibodies used were the following: p-TrkB (Abcam), Her2 (Life Technologies), BDNF (Abcam), and GFAP (Millipore).

Western Blot Analysis

Cells were centrifuged (425 x g, 5 min) and then homogenized in lysis buffer (Glycerol [Sigma], 3 M KCl [Sigma], 10% NP-40 [Sigma], 1 M Tris [Bio-Rad], and 1 M

DTT [Sigma]) containing phosphatase inhibitor (ThermoScientific), EDTA (ThermoScientific), and protease inhibitor (ThermoScientific) for 15 min. Samples were then centrifuged (13362 x g, 10 min), and the supernatant protein was collected. Protein (40 μg) was boiled for 5 min with loading buffer (laemmli [Bio-Rad], β-mercaptoethanol [Bio-Rad]) and loaded onto mini-protean TGX gels (Bio-Rad). The gel was run at 200 V then transferred onto a membrane at 100 V for 1 h. After the presence of protein was confirmed by Ponceau staining, the membrane was blocked for 1 h with blocking buffer (ThermoScientific). Primary antibody diluted in blocking buffer was applied overnight at 4°C. The following day, horseradish peroxidase (HRP)-conjugated secondary antibody (ThermoScientific) was applied for 1 h at room temperature. Membranes were exposed to Supersignal West Pico Chemi-luminescent Substrate (ThermoScientific) for 5 min and then scanned using a Li-Cor c-Digit membrane scanner (Li-Cor). Antibodies used were as follows: p-TrkB (Abcam), Her2 (Life Technologies), p-PI3K p55/p85 (Cell Signaling Technology), p-AKT (Cell Signaling Technology), BDNF (Abcam), and β-actin (Cell Signaling Technology).

Immunocytochemistry

Cell cultures were fixed on collagen-coated cover slips (Warner Instruments) with 4% paraformaldehyde (Electron Microscopy Services) at room temperature for 15 min. Cells were permeabilized in a 0.3% Triton X-100 solution with PBS for 45 min at 37°C, and blocked for 1 h in a blocking solution composed of 10% NGS, 1% BSA, and 10% glycine. Subsequent steps are described in the immunohistochemistry methods. Refer to the previous section for a list of antibodies used in these experiments.

Flow Cytometry

Cell cycle analysis of cell incubated with or without BDNF was analyzed by propidium iodide staining (PI). Control cells or cells treated with 25 ng/mL BDNF for 5 days were collected and fixed with 70% ethanol. Before flow cytometry analysis, cells were resuspended in PI solution (1 mg/mL propidium iodide dye [Life Technologies], 0.1% Triton-X and 2 mg DNase-free RNase A in PBS) overnight. Cells were quantified and analyzed with CyAn.

Populations of marker-positive cells were quantified by flow cytometry analysis. One million cells/sample were stained with Live/Dead Fixable Aqua Staining (Life Technologies) for 30 min on ice. Pellets were fixed with 3.7% formaldehyde (Electron Microscopy Services) for 15 min at room temperature. Primary antibodies were applied for 2 h at room temperature and fluorescent secondary antibodies were applied for 1 h in the dark at room temperature. The presence of markers on cells was detected and quantified by BD Fortessa. Antibodies used were Her2 (Novus Biologicals) and TrkB (Cell Signaling Technology).

Enzyme-Linked Immunosorbent Assay (ELISA)

Media from cultures of BBM1 cells, 361 cells, astrocytes, and fibroblasts were collected, centrifuged (425 x g), and frozen. Cells were counted to quantify the amount of BDNF released by each cell. A 96-well plate coated with BDNF antibody in an ELISA kit (R&D systems) was used to measure BDNF in media. Samples were measured at 450 nm and the amount of BDNF released was quantified as femtograms released per cell.

Computational Methods for Generating a Structural Model of the Her2-TrkB Complex

The monomer structures of Her2 (pdb_ID 3PP0) (28) and TrkB (pdb_ID 4ASZ) (29) were selected for use as the input structures. Hydrogen atoms and missing side chains were added to the crystal structures using the Protein Preparation Wizard (30) in the Maestro 9.1 suite (31). The structures were then minimized to repack the hydrogen atoms, constraining all of the backbone atoms. The minimized structures were then docked as rigid bodies using the ZDOCK server (32). To evaluate the top 5 models obtained from the Zdock server, side chain orientations of residues in the interface were first optimized using Prime and then the energy of each model was minimized (33, 34). The resulting structures were evaluated by attempting to maximize the following criteria: (i) binding energy of the complex, (ii) interaction surface between the two proteins, and (iii) the number of favorable electrostatic interactions, hydrophobic amino acid interactions, and hydrogen bonds between the docked proteins. The best scoring complex was also compared to the Her2 Her3 heterodimer structure as described in (35). Using these criteria we selected the most favorable complex and displayed the possible side-chain side-chain hydrogen bonds. The resulting figure was generated using PyMOL viewer (36).

Electron Microscopy

Control BBM1 cells or BBM1 cells treated with 25 ng/mL BDNF were collected and cryo-fixed in a high pressure freezer. Sections (~70 nm thick) were cut using a

Leica Ultra cut UCT ultramicrotome with a diamond knife and placed on mesh nickel electron microscope grids. Grids were stained with 2% uranyl acetate in 70% ethanol for 1 min followed by Reynold's lead citrate staining for 1 min. For post-embedding immuno-labeling, antigens were detected with 10 nm (for TrkB) or 20 nm (for Her2) colloidal gold conjugated secondary antibody. For pre-embedding immuno-labeling, cells were incubated with TrkB antibody overnight and then incubated with secondary antibody conjugated with 1.4 nm nanogold at room temperature. Cells were fixed (0.2% glutaraldehyde in PBS, room temperature, 10 min) and developed for 5 min with an HG silver enhancement kit (Nanoprobes). After the cell pellet was sectioned, post-embedding labeling was performed with the Her2 antibody overnight. Labeled sections were incubated with colloidal gold conjugated secondary antibody (15 nm) for 1 h. Images were acquired at 11000x magnification. Cells were imaged and receptors were quantified by analyzing 200 images per receptor (100 images from the pre-embedding method and 100 images analyzed from the post-embedding method).

Co-Immunoprecipitation

Cells were homogenized (15 min, on ice) in lysis buffer (see western blot methods) containing protease inhibitor, EDTA, and phosphatase inhibitor. Proteins were harvested by centrifugation (13362 x g, 10 min, 4°C). Protein (750 μg) and antibody (4 μg) conjugated with Protein G agarose beads (Invitrogen) were incubated overnight at 4°C with agitation. The next day, tubes were centrifuged (7500 x g, 30 s) and washed 4 times. Pellets were resuspended in loading buffer (95% Laemmli Buffer, 5% β-mercaptoethanol) and loaded onto Mini-PROTEAN TGX Gels (Bio-Rad). Protein bands

were incubated with appropriate primary antibodies overnight at 4°C. Membranes were incubated with horseradish peroxidase (HRP)-conjugated secondary antibodies the following day at room temperature for 1 h, and bands were detected by Supersignal West Pico Chemiluminescent Substrate. Membranes were imaged by Li-Cor scanning (Li-Cor). Antibodies used were Her2 (Life Technologies) and TrkB (Abcam).

Cell Viability Assays

For cell viability assays, 2000 cells were plated per well (day 0) in a 96 well plate. Twenty-four hours later, cells were either left untreated or were treated with cyclotraxin B (10 μM) alone, cyclotraxin B (10 μM) + Lapatinib (1 μM) or cyclotraxin B (10 μM) + Lapatinib (1 mM) + BDNF (50 ng/mL). Each sample was run in triplicate. Cells were stained with crystal violet solution (0.05% crystal violet, 1% formaldehyde, 1% methanol, 1x PBS, in ddH_20) for 10 min at room temperature. The stain was then solubilized with 1% SDS before samples were read at 570 nm.
