# Supplementary material for: Cooperation of neurotrophin receptor TrkB and Her2 in breast cancer cells facilitates brain metastases
Source: Breast Cancer Res. 2017 Apr 26;19:51. doi: 10.1186/s13058-017-0844-3 (PMC5406906; doi:10.1186/s13058-017-0844-3)
Supplement: Supplementary file 2 — Expression of TrkB in various cells and tissue. Figure S2. Analysis of TrkB knockdown with two different TrkB shRNAs in patient-derived MDA-MB-361 cells (361-KD and 361-KD2). Figure S3. Western blot analysis of protein extracts from patient-derived 361 cells and 361 cells transduced with TrkB shRNA-1 (361-KD) or TrkB shRNA-2 (361-KD2) and cultured with BDNF. Figure S4. Real-time PCR quantification of BDNF expression in different metastasis sites. Figure S5. Western blot analysis of intracellular BDNF in BBM1 cells, 361 cells, astrocytes, and fibroblasts and proliferation of 361 cells and TrkB-specific shRNA1 (361-KD) and shRNA2 (361-KD2) transduced cells grown in astrocyte or fibroblast conditioned medium (CM). Figure S6. TrkB knockdown disrupts the metastatic efficiency of BBM2 xenografts. Figure S7. TrkB knockdown disrupts colonization and metastatic efficiency in BBM xenografts. Figure S8. TrkB and Her2 are co-expressed in BBM and breast cancer cells. Figure S9. Immunocytochemical staining of BBM1 and TrkB knockdown (BBM1-KD) cells treated with BDNF over different times. (PDF 1352 kb) [file 13058_2017_844_MOESM2_ESM.pdf]

Supplementary Figures, Choy et al.

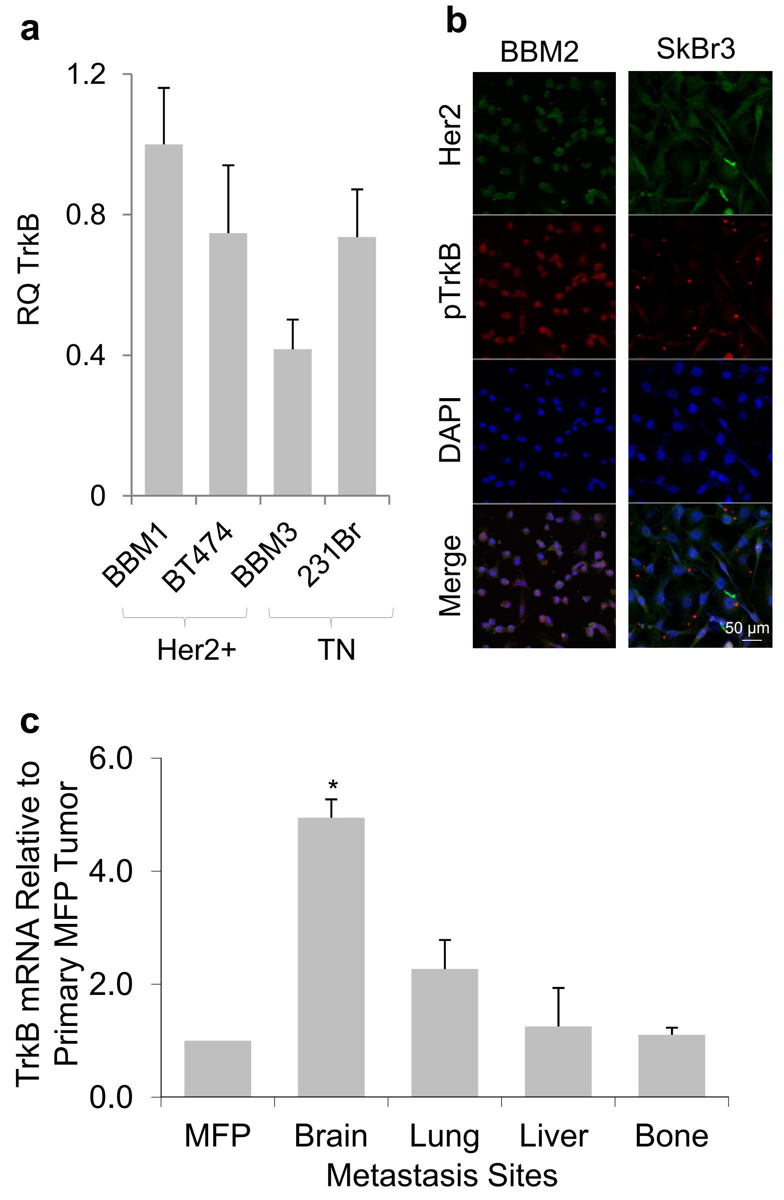

Fig. S1: Expression of TrkB in various cells and tissue. (**a**) Real-time PCR quantification of TrkB levels in BBM1, BT474, BBM3, and MDA-MB-231 cells (n = 4). Bars indicate SEM. (**b**) Immunofluorescence staining of pTrkB in BBM2 (brain metastases) and SkBr (breast cancer) cells with antibodies to Her2 (green) and phosphorylated TrkB (pTrkB, red). Nuclear counter staining was done with DAPI (blue). (**c**) Real-time PCR quantification of TrkB mRNA levels in brain metastasis, lung metastasis, bone metastasis, and liver metastasis tissue relative to the primary MFP tumor site. BBM1 cells were injected in the mammary fat pad and metastatic sites were dissected and processed to extract mRNA. mRNA was used for real time PCR quantification of TrkB. GAPDH and actin were used as controls. *$p < 0.05$. Bars indicate SEM.

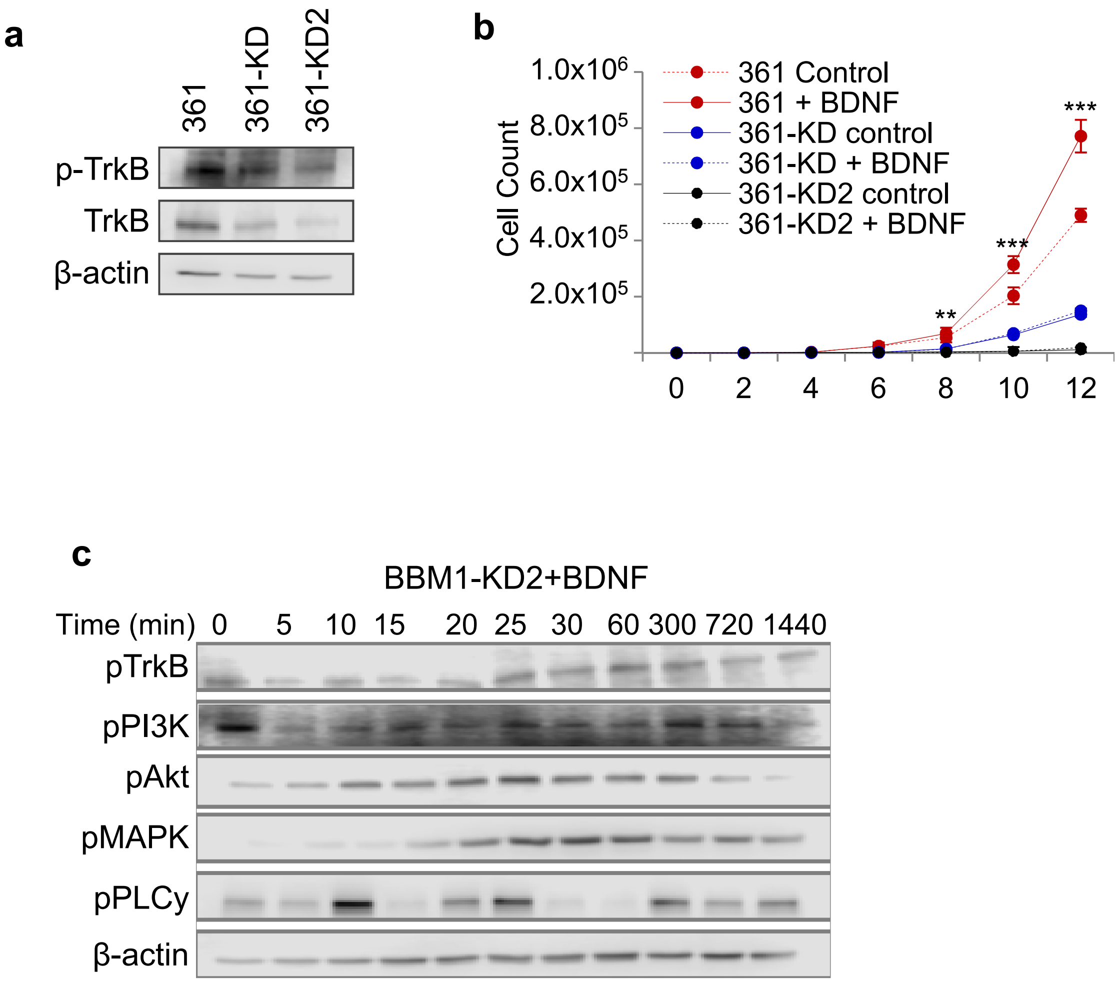

Fig. S2: (**a**) Western blot analysis of TrkB knockdown with two different TrkB shRNAs in patient-derived MDA-MB-361 cells (361-KD and 361-KD2). Control cells were transected with empty vector. (**b**) Effect of exogenous BDNF (25 ng/mL) on proliferation of patient-derived 361 cells over 12 days *in vitro*. ****p < 0.0001. Bars indicate SEM. (**c**) Western blot analysis of protein extracts of TrkB shRNA-2 expressing BBM1 (BBM1-KD2) cells cultured with BDNF (25 ng/mL) for the indicated times. β-actin was used as a loading control.

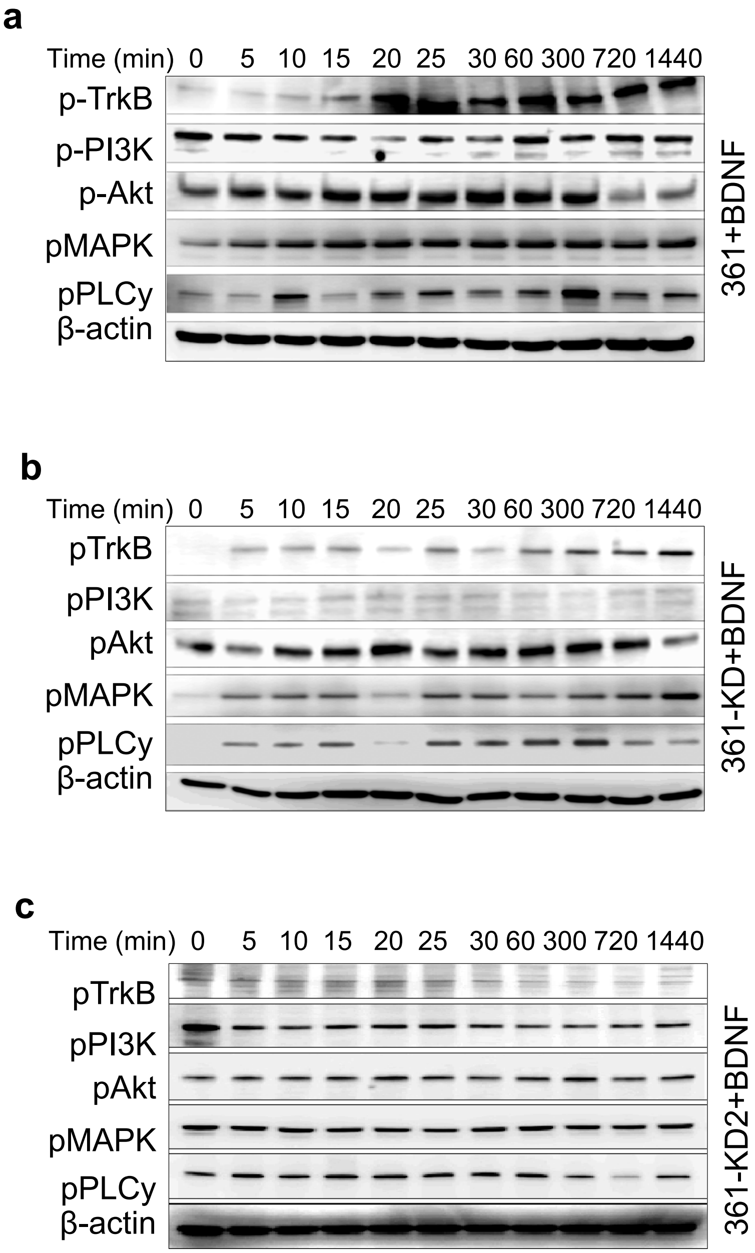

Fig. S3: (**a**-**c**) Western blot analysis of protein extracts from patient-derived 361 cells and 361 cells transduced with TrkB shRNA-1 (361-KD) or TrkB shRNA-2 (361-KD2) and cultured with BDNF (25 ng/mL) for the indicated times. β-actin was used as a loading control.

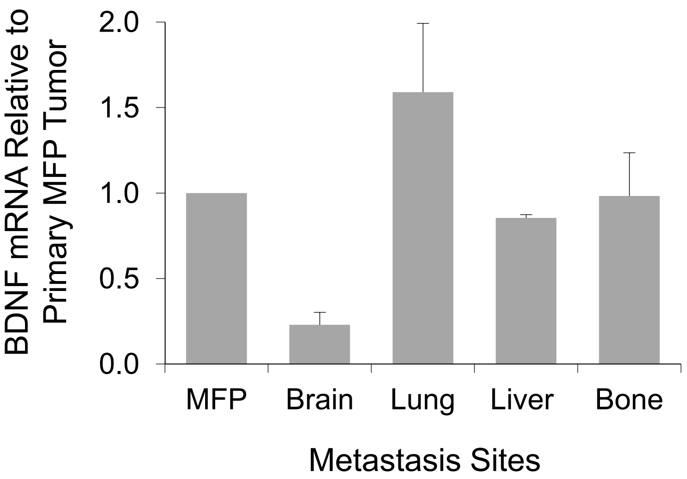

Fig. S4: BBM1 cells were injected in mammary fat pad (MFP) of NOD-SCID mice and followed for 35 days. After mice were euthanized, the metastatic sites (brain, lung, liver, and bone) were dissected and processed for mRNA extraction. mRNA was used to perform real time PCR quantification of BDNF expression. GAPDH and actin were used as controls. n = 6, Bars indicate SEM.

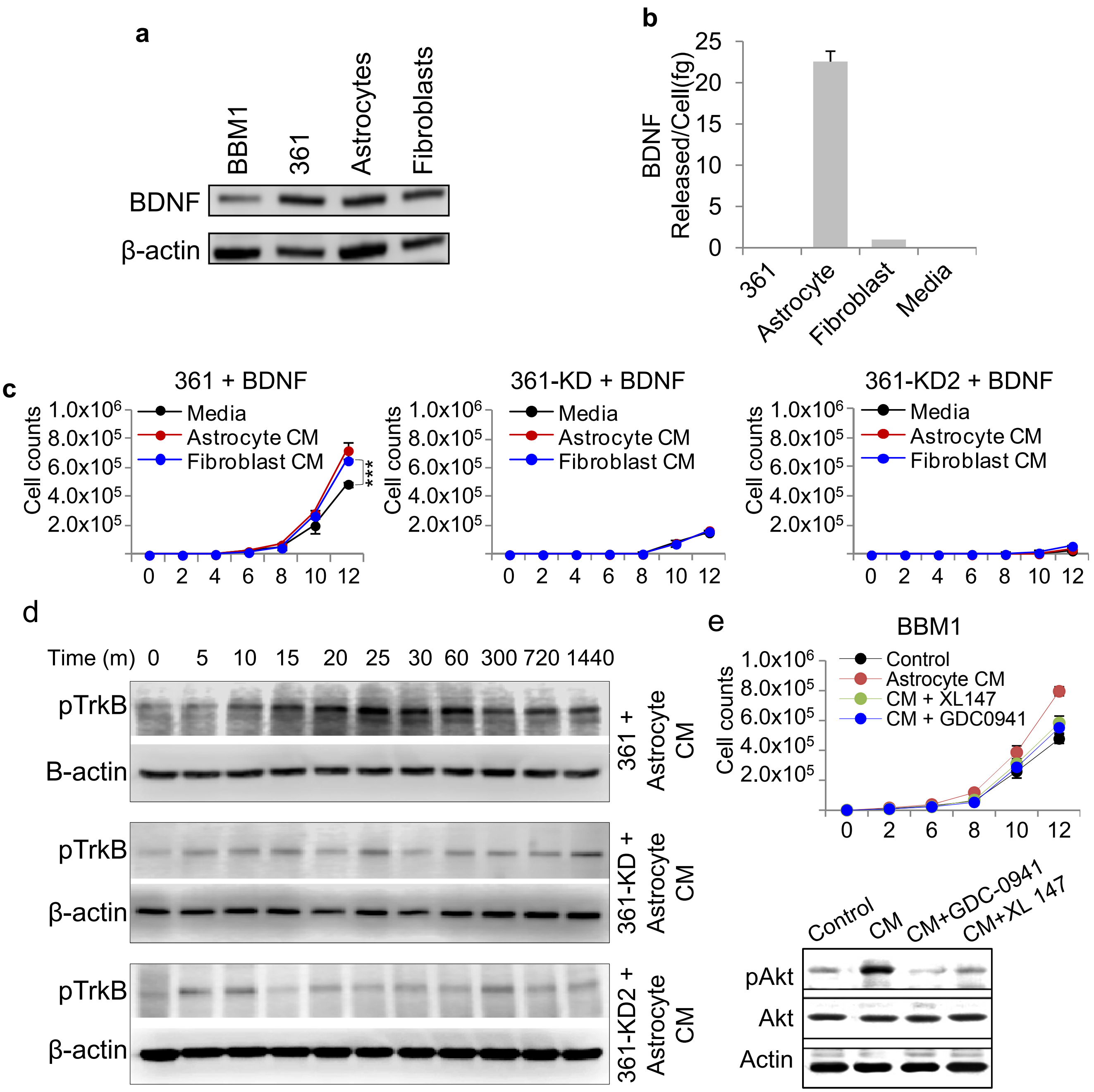

Fig. S5: (**a**) Western blot analysis of intracellular BDNF in BBM1 cells, 361 cells, astrocytes, and fibroblasts. β-actin was used as a loading control. (**b**) ELISA quantification of BDNF released from patient-derived 361 cells, astrocytes, and fibroblasts as compared to media alone. ****$p<0.0001$. Bars indicate SEM (n = 8). (**c**) Proliferation of 361 cells and TrkB-specific shRNA1 (361-KD) and shRNA2 (361-KD2) transduced cells grown in astrocyte or fibroblast conditioned media (CM) for the indicated time periods. Control cells were transduced with empty vectors. Media alone was used as negative control. ***$p<0.001$, ****$p<0.0001$. Bars indicate SEM (n = 6). (**d**) Western blot analysis of protein extracts from patient-derived 361 cells and 361 cells transduced with TrkB shRNA-1 (361-KD) and TrkB shRNA-2 (361-KD2) cultured in astrocytes CM for the indicated times. β-actin was used as a loading control. (**e**) Proliferation of BBM1 cell grown in astrocyte conditioned media (CM) containing PI3K inhibitor XL147 and GDC0941 (1μM) (CM) for the indicated time periods (top panel). Inhibition of PI3K signaling was confirmed by Western blot analysis of Akt and pAkt (bottom panel). Actin was used as loading control.

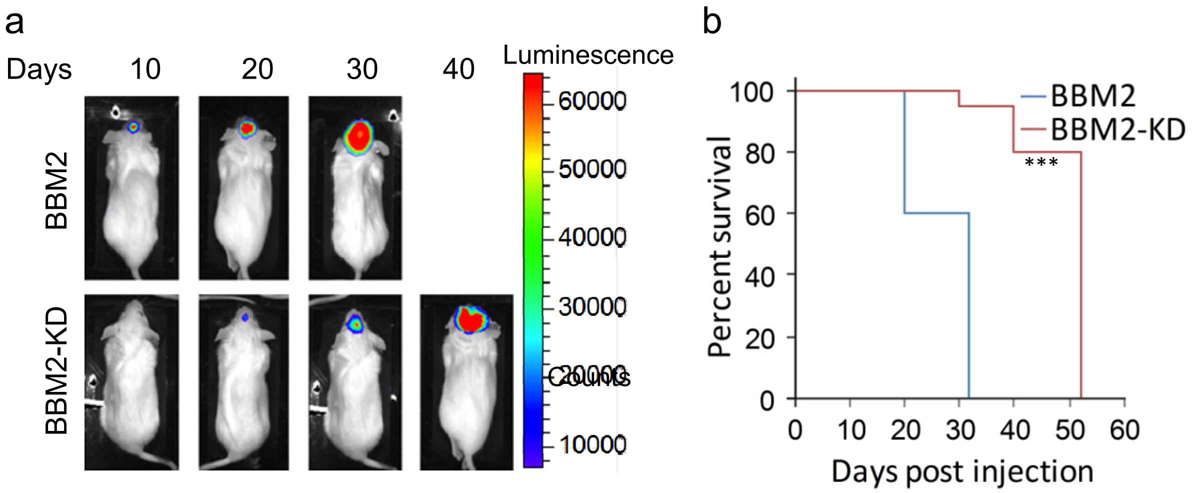

Fig. S6: TrkB knockdown disrupts metastatic efficiency of BBM2 xenografts. (**a**) Representative bioluminescence imaging (BLI) of intracranially injected BBM2 or BBM2-KD cells in NOD-SCID mice. (**b**) Survival curve of mice injected intracranially with BBM2 or BBM2-KD cells. ***p<0.01 (n=12).

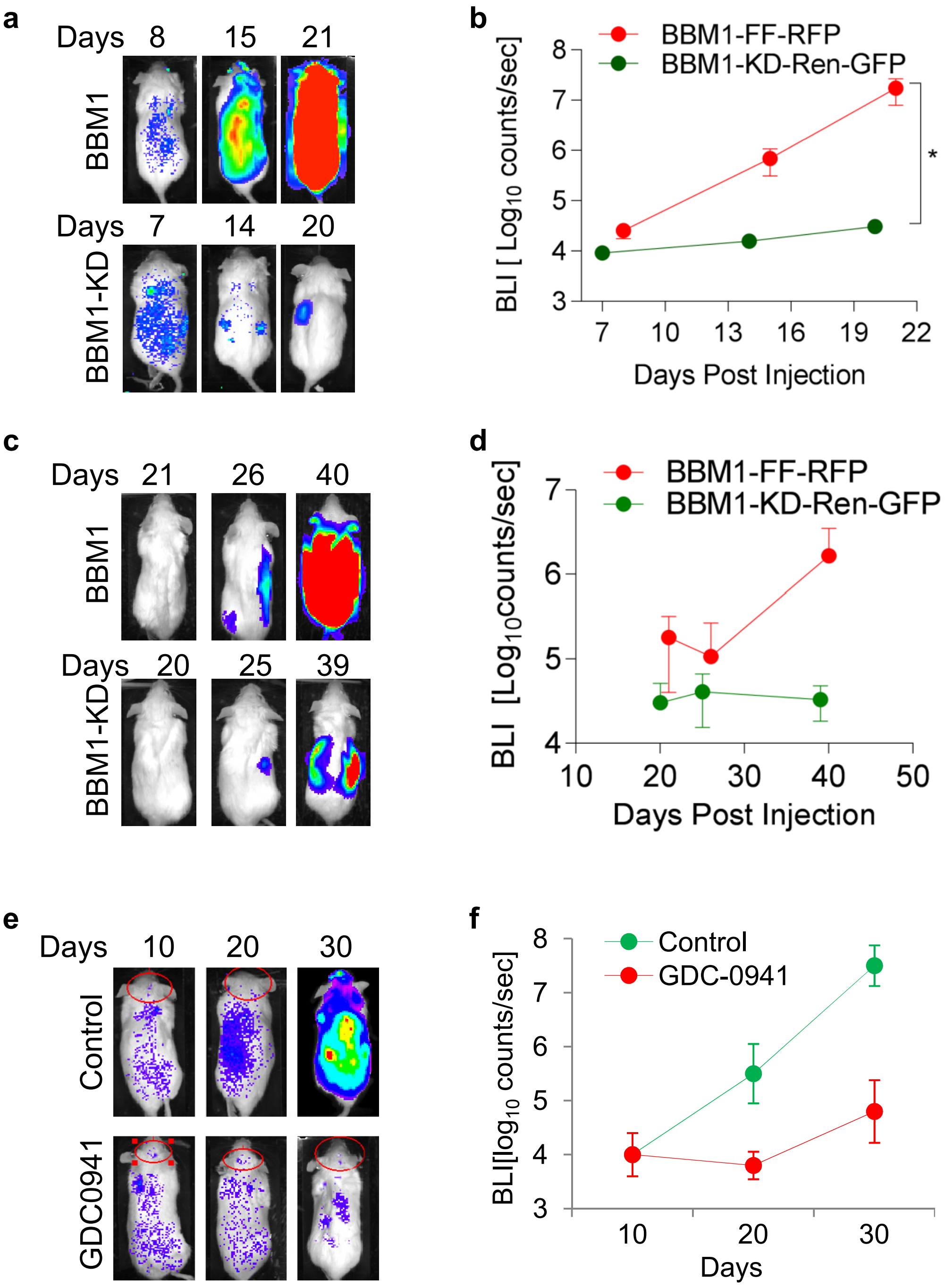

Fig. S7: TrkB knockdown disrupts colonization and metastatic efficiency in BBM xenografts. (**a**) Representative bioluminescence imaging (BLI) of NOD-SCID mice that received intracardiac injections of BBM1-FF-RFP (top) and BBM1-KD-Ren-GFP (bottom) cells at a 1:1 ratio. (**b**) Quantification of BLI intensity (p/sec/cm^2/sr) of brain metastases in mice from **a**. (**c**) Representative BLI of NOD-SCID mice that received mammary fat pad MFP injected BBM1-FF-RFP (top) and BBM1-KD-Ren-GFP (bottom) cells (1:1 ratio). (**d**) Quantification of BLI intensity (p/sec/cm^2/sr) of brain metastases in mice from **c**. (**e**) Representative bioluminescence imaging (BLI) of NOD-SCID mice that received intracardiac injections of BBM1-FF-RFP cells followed by oral dosing of GDC-0941 at 5 days interval. (**f**) Quantification of BLI intensity (p/sec/cm^2/sr) of metastases in mice from **e.**

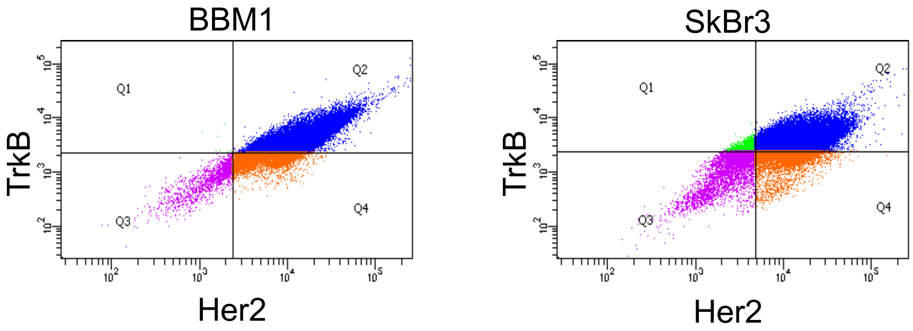

Fig. S8: TrkB and Her2 are coexpressed in BBM and breast cancer cells. Flow cytometry of Her2 and TrkB expression in BBM1 and SkBr3 cells.

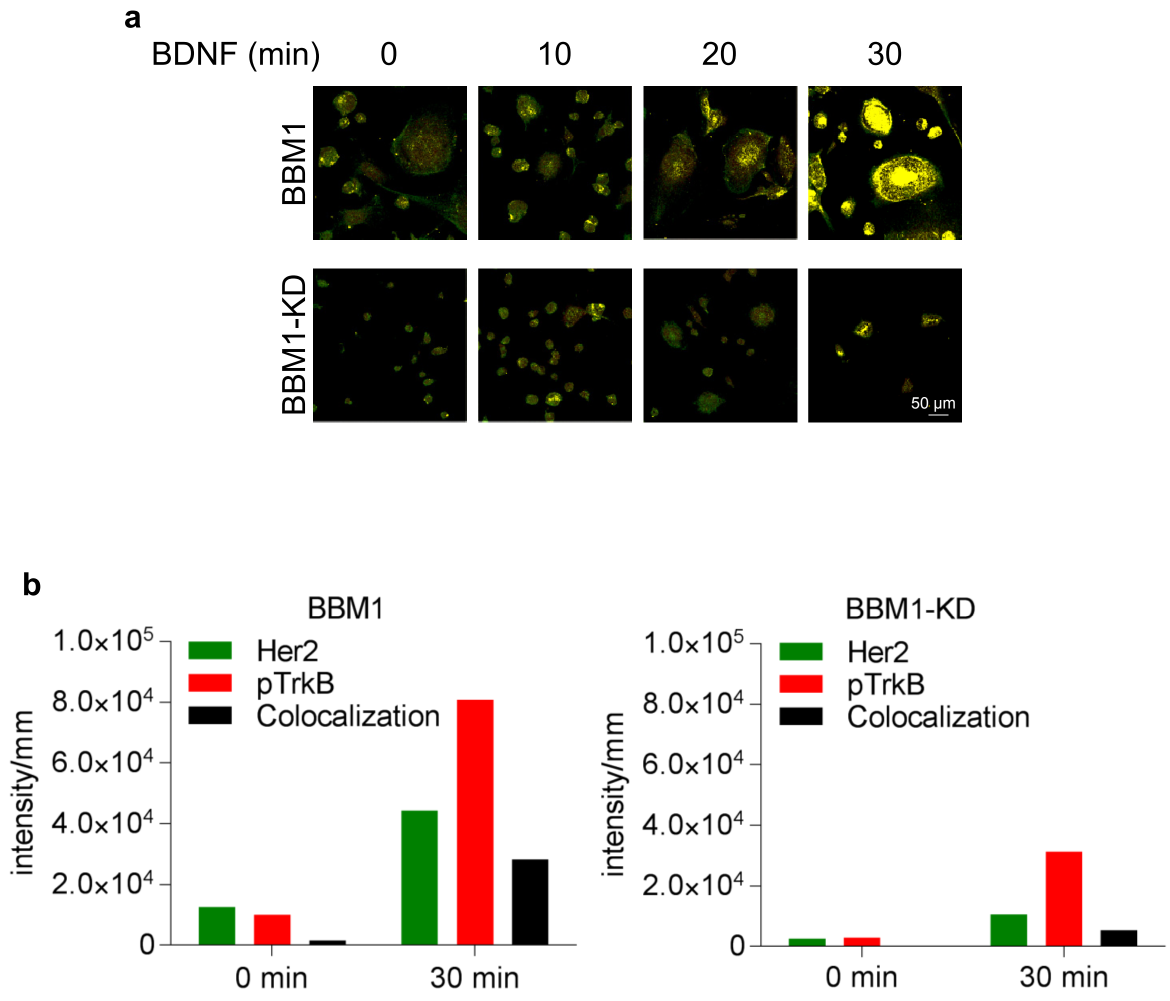

Fig. S9: (**a**) Immunocytochemical staining of a 30 minute time course for BBM1 and TrkB knockdown (BBM1-KD) cells treated with BDNF (25 nM). Yellow indicates colocalization. (**b**) Quantification of Her2 and TrkB and co-localization (intensity) over time in BBM1 and BBM-KD cells.
